# Light Is More Important Than Nutrient Ratios of Fertilization for *Cymodocea nodosa* Seedling Development

**DOI:** 10.3389/fpls.2018.00768

**Published:** 2018-06-13

**Authors:** Ana Alexandre, João Silva, Rui Santos

**Affiliations:** Marine Plant Ecology Research Group, Centre of Marine Sciences (CCMAR), University of Algarve, Faro, Portugal

**Keywords:** *Cymodocea nodosa*, germination, light, nutrient ratios, nutrient uptake, seeds, seedlings

## Abstract

Restoration of seagrass beds through seedlings is an alternative to the transplantation of adult plants that reduces the impact over donor areas and increases the genetic variability of restored meadows. To improve the use of *Cymodocea nodosa* seedlings, obtained from seeds germinated *in vitro*, in restoration programs, we investigated the ammonium and phosphate uptake rates of seedlings, and the synergistic effects of light levels (20 and 200 μmol quanta m^-2^ s^-1^) and different nitrogen to phosphorus molar ratios (40 μM N:10 μM P, 25 μM N:25 μM P, and 10 μM N:40 μM P) on the photosynthetic activity and growth of seedlings. The nutrient content of seedlings was also compared to the seed nutrient reserves to assess the relative importance of external nutrient uptake for seedling development. Eighty two percent of the seeds germinated after 48 days at a mean rate of 1.5 seeds per day. All seedlings under all treatments survived and grew during the 4 weeks of the experiment. Seedlings of C. *nodosa* acquired ammonium and phosphate from the incubation media while still attached to the seed, at rates of about twice of adult plants. The relevance of external nutrient uptake was further highlighted by the observation that seedlings’ tissues were richer in nitrogen and phosphorus than non-germinated seeds. The uptake of ammonium followed saturation kinetics with a half saturation constant of 32 μM whereas the uptake of phosphate increased linearly with nutrient concentration within the range tested (5 – 100 μM). Light was more important than the nutrient ratio of fertilization for the successful development of the young seedlings. The seedlings’ photosynthetic and growth rates were about 20% higher in the high light treatment, whereas different nitrogen to phosphorus ratios did not significantly affect growth. The photosynthetic responses of the seedlings to changes in the light level and their capacity to use external nutrient sources showed that seedlings of *C. nodosa* have the ability to rapidly acclimate to the surrounding light and nutrient environment while still attached to the seeds. *C. nodosa* seedlings experiencing fertilization under low light levels showed slightly enhanced growth if nourished with a balanced formulation, whereas a slight increase in growth was also observed with unbalanced formulations under a higher light level. Our results highlight the importance of high light availability at the seedling restoration sites.

## Introduction

Seagrass meadows are invaluable marine habitats considering the myriad of goods and services they provide to the overall functioning of coastal systems. Seagrass beds increase biodiversity, offer food and shelter for marine animals, improve water quality, protect coastlines from erosion and mitigate climate change through carbon storage (Duffy, 2006; Fourqurean et al., 2012). Despite their ecological importance, seagrasses are a vulnerable resource. Concern is increasing over the decline of seagrass populations worldwide as a result of adverse anthropogenic activities in coastal areas (Orth et al., 2006a; Short et al., 2011).

Seagrass restoration programs have long been used in an attempt to re-establish lost or declining seagrass areas. Seagrass restoration involves the transplantation of plant material (mature plants or seeds and seedlings from *in vitro* germinated seeds) obtained from healthy donor meadows into degraded areas. Recently, small- and large-scale restoration efforts have been made to recover lost populations in Northern Europe using both vegetative transplants (van Katwijk et al., 1998) and seed-based methods (Infantes et al., 2016), in Chesapeake Bay, United States (Orth et al., 2003, 2006b, 2009, 2012) and in Shandong Peninsula, China (Zhang et al., 2015; Yang et al., 2016) using seed-based methods, and in Western Australia using vegetative transplants (Paling et al., 2001, 2007; van Keulen et al., 2003; Bastyan and Cambridge, 2008). Transplantation of mature plants harvested from donor populations, frequently located a great distance from receptor meadows, requires a heavy logistic and creates an impact on donor meadows. The survival of the transplants at the receptor sites has been highly variable among the various projects. Compared to the transplantation of adult plants, the use of seeds (or *in vitro* germinated seedlings) in seagrass restoration constitutes an effective alternative, or complementary, re-plantation method that reduces the impact over donor areas, requires less labor and time, is less expensive, and increases the genetic variability within restored meadows (Ackerman, 2006; Ailstock and Shafer, 2006; Zarranz et al., 2010). Genetically diverse seagrass populations grow and spread faster, produce more flowers, and have better rates of germination than less diverse beds (Procaccini and Piazzi, 2001; Williams, 2001). Therefore, seed- and seedling-based restoration has been considered more suitable for large-scale seagrass restoration projects (Williams and Orth, 1998; Harwell and Orth, 1999; Granger et al., 2000; Orth et al., 2008). However, this restoration practice presents several bottlenecks (Statton et al., 2017), one of them being the low rate of seedling survival and establishment (less than 10% of the seeds placed in the field) (Orth and Moore, 1983; Orth et al., 2003), although high rates of success have been also observed (Balestri et al., 1998; Balestri and Lardicci, 2012). Due to the vulnerability of seedlings to environmental stressors during the initial phases of development, the successful development of viable beds depends upon several factors, including sediment burial, light, nutrients and initial patch size (Duarte and Sand-Jensen, 1990; Moore et al., 1993; Celdrán and Marín, 2013).

*Cymodocea nodosa* is a subtidal dioecious marine angiosperm that forms extensive meadows along the Mediterranean coasts, the North Atlantic coast of Africa and the Canary Islands, reaching its southern limit of distribution in Senegal (Pavón-Salas et al., 2000, Green and Short, 2003; Alberto et al., 2008; Cunha and Araújo, 2009). It is the most common seagrass species in the Canary Islands (Reyes et al., 1995) and the second most important concerning the surface area covered in the Mediterranean, after *Posidonia oceanica* (Procaccini et al., 2003). The species can reproduce both clonally and sexually through the production of flowers, fruits and seeds that originate seedlings (Caye and Meinesz, 1985; Buia and Mazzella, 1991; Terrados, 1991). Substantial deterioration and regression of *C. nodosa* meadows has occurred in the past two decades (Delgado et al., 1997; Tuya et al., 2013, 2014; Fabbri et al., 2015). Efforts have been made to restore seagrasses by transplanting adult plants from donor to receptor meadows (Curiel et al., 2003; Meinesz et al., 2005; Ruiz de la Rosa et al., 2006) but so far no cases of successful *C. nodosa* seedlings transplantations are reported. Little is known about the environmental factors controlling the development of seedlings and its establishment, although this information is key for the success of restoration programs through seedlings. Nutrient limitation at the seedling stage has been shown to constrain branching and patch initiation in *C. nodosa* (Duarte and Sand-Jensen, 1996; Nielsen and Pedersen, 2000; Balestri et al., 2010), suggesting that nutrients play a critical role in the successful establishment of replanted seedlings in nutrient-poor environments. Therefore, the application of fertilizers in transplanted areas has been proposed to minimize nutrient limitation and enhance the survival and growth of the seedlings at the initial stages of development. A number of fertilization experiments have shown that the addition of nitrogen and phosphorus to seagrass meadows stimulates shoot biomass and growth of *C. nodosa* (Pérez et al., 1991, 1994; Kenworthy and Fonseca, 1992; Balestri et al., 2010; Balestri and Lardicci, 2014), *P. australis* and *P. sinuosa* (Hovey et al., 2012), and seedlings of *Zostera marina* (Tanner and Parham, 2010) but not in seedlings of *P. australis* (Statton et al., 2014). Nutrient additions to seagrass meadows have usually been made through the application of fertilizers with either balanced or unbalanced nutrient formulations. The effects of the application of balanced and unbalanced formulations of fertilizers (i.e., different N and P molar ratios) on growth and shoot production of seagrass seedlings have been evaluated in only two studies (Roberts et al., 1984; Tanner and Parham, 2010). Both studies reported enhanced growth rates of seedlings fertilized with a balanced formulation. However, nitrogen to phosphorus molar ratios in seagrass meadows are seldom balanced, and interactions between ammonium and phosphate uptake have been reported. While, on the one hand, the addition of phosphate has been shown to attenuate the negative production balance of the seagrass *Z. noltei* nourished with high concentrations of ammonium at low light levels (Brun et al., 2008), on the other hand an inhibition of phosphate uptake in the presence of ammonium has been described in this species (Villazán et al., 2013). Based on these observations, we tested different N to P molar ratios and hypothesized whether a balanced or an unbalanced nutrient formulation would result in a higher growth rate of the *C. nodosa* seedlings. Light reduction, mostly due to anthropogenic disturbances, is a major cause of seagrass die-off in shallow coastal areas (Short and Wyllie-Echeverria, 1996). Seedlings are particularly sensitive to light availability, as its survival, growth and development is significantly affected by light reduction (Bintz and Nixon, 2001). Light and nutrient regimes are frequently reported as major factors affecting seagrass seedling development and the success of restoration efforts.

In order to improve the use of seedlings in *C. nodosa* restoration programs, we assessed (1) the seedling nitrogen and phosphorous uptake dynamics and (2) the synergistic effects of light and N:P ratios on photosynthesis and growth in lab-developed seedlings. The seed development and germination rates were also assessed. To determine the relative importance of external nutrient uptake for seedling development we also followed the nutrient content of non-germinated seeds and young seedlings. We hypothesize that seedlings’ growth will be enhanced under high light level and fertilization with a balanced nutrient formulation.

## Materials and Methods

### Germination of Seeds

Seeds of *Cymodocea nodosa* were collected from the sediment of a meadow at the southeast coast of Gran Canaria Island, Spain (27°47′19″N, 15°29′35″W) in November 2015 and were transported in moist tissue to the Centre of Marine Sciences, South Portugal. Seeds were induced to germinate by the hyposaline shock (Caye et al., 1992) in a growth chamber in aquaria with natural seawater (salinity = 18‰, pH = 8.2, N and P < 1 μM) semi-buried in autoclaved (20 min at 120°C) sea sand at 22°C with a light intensity of 150 μmol quanta m^-2^ s^-1^ and a photoperiod of 12:12 h. A salinity of 18‰ was chosen for the hyposaline shock because it was reported to yield higher percentages of seed germination and seedlings with leaves in *C. nodosa* (Zarranz et al., 2010). After seeds were germinated, i.e., after the emergence of the cotyledon, seeds were transferred to aquaria with full-strength natural seawater (salinity = 36‰, pH = 8.2, N and P < 1 μM) to ensure a higher percentage of developed seedlings (Zarranz et al., 2010). The seedlings’ development was followed from seed germination (visible cotyledon) to seedling production (plant with young leaves and roots still attached to the seed) and the percentage of seeds in the different developmental stages was recorded (*n* = 90). The mean germination rate was also determined.

Seeds of *C. nodosa* were also collected from the sediment of a meadow in Laguna Mar Menor, Múrcia, Spain (37°49′08″N, 0°46′33″W) in a dense monospecific meadow at 0.6 m depth in November 2015, but only 2% of the seeds germinated under the same germination conditions used for the seeds collected in Gran Canaria, demonstrating that the germination potential is site specific. Given the low germination rate of the seeds from Mar Menor, the experiments were performed using only the seedlings from seeds collected in Gran Canaria. Seedlings with at least two developed leaves and roots still attached to the seed (about 2-month old) were used.

### Uptake Experiments

In a first experiment, seedlings were incubated for 0.5 h in 250 mL of artificial seawater (salinity = 35‰, pH = 8.2) enriched with ^15^NH_4_Cl (at % = 99) at five nutrient concentrations (5, 25, 50, 100, and 200 μM). For each nutrient concentration eight seedlings were incubated, in a total of 40 seedlings (8 seedlings × 5 concentrations). Each seedling was incubated in an independent container. A short incubation time (0.5 h) was chosen to ensure that nutrient concentrations in the media remained constant throughout the incubation, and that the incorporation of the labeled nutrient by plant tissues was not affected by changes in the nutrient concentration. In a second experiment, seedlings were incubated for 4 h in 100 ml of artificial seawater (salinity = 35‰, pH = 8.2) enriched with K_2_HPO_4_ at five different nutrient concentrations (5, 12.5, 25, 50, and 100 μM). For each nutrient concentration five seedlings were incubated, in a total of 25 seedlings (5 seedlings × 5 concentrations). Each seedling was incubated in an independent container. In this experiment, uptake rates were assessed using the depletion method, i.e., rates were determined by the difference between the initial and final concentration of the nutrient in the media and, thus, a longer incubation time (4 h) and a shorter incubation volume (100 ml) were required.

Experiments were performed at constant light (125 μmol quanta m^-2^ s^-1^) and temperature (22°C). During the experiments, the seedlings were incubated in seawater only, after being grown in autoclaved sea sand. The incubation media were constantly stirred using a shaking platform to ensure a thorough mixing of the nutrients. The range of nutrient concentrations used was representative of seagrass environments (Touchette and Burkholder, 2000). Although nutrient concentrations in the water column are much lower (<5 μM) compared to those in the sediment porewater, pulses of high nutrient concentration may also occur from the sediment to the water column in seagrass meadows (e.g., up to 200 μM in the Palmones river estuary during low tide, Brun et al., 2002). The dry biomass (leaves plus roots) of the incubated seedlings in the N and P uptake experiments averaged 0.028 ± 0.013 g and 0.027 ± 0.010 g, respectively.

At the end of the N uptake experiment, the leaves and roots of the seedlings were separated from the seeds, dried at 60°C for 48 h and weighted. The dried tissues were reduced to a fine powder for ^15^N analysis. The amount of ^15^N (g) taken up by the leaves and roots of the seedlings was calculated by subtracting the post-incubation ^15^N level (%) from the initial background level (%) and multiplied by the total nitrogen in the tissues (g). Nitrogen uptake rates (μmol g^-1^ DW h^-1^) were plotted against substrate concentration (μM) and fitted to the Michaelis–Menten model (V = V_max_ × S/K_m_ + S), and the uptake kinetic parameters V_max_ (maximum uptake rate), K_m_ (half-saturation constant) and α (affinity constant = V_max_/K_m_) were obtained. S is the substrate concentration (μM).

At the end of the P uptake experiment, the seedlings were removed from the incubation media, dried at 60°C for 48 h and weighted. Water samples were collected from each seedling container, filtered (Whatman cellulose acetate filters, 0.45 μm size pore) and stored at -20°C until analysis. The concentration of phosphate in the samples was measured in a loop-flow analyser (μMAC-1000, Systea, Anagni, Italy) using the molybdate/ascorbic acid method. Phosphate uptake was determined as the difference between the initial and final concentration of the nutrient in the seawater. Uptake rates were fitted to a linear model V = a + b × S, where S is the substrate concentration (μM).

### Nutrient Content and Requirements for Growth

The endosperm of non-germinated seeds and the leaves and roots of developed seedlings still attached to the seed (*n* = 5), were dried and grinded into powder for determination of the carbon (C), nitrogen (N) and phosphorus (P) content to follow the nutrient content throughout developmental phases. C and N analysis were performed in a Vario EL III elemental analyser (Elementar). P content was determined by alkaline persulfate digestion following the methods described by Murphy and Riley (1962) and Wheeler and Björnsäter (1992). Briefly, 6 mL of oxidizing agent (3 g sodium hydroxide and 6.7 g potassium persulfate in 1 L of distilled water) were added to 1 – 2 mg of ground dried tissue (60°C for 48 h) and autoclaved at 120°C for 1 h. After cooling, samples were acidified with 0.6 mL of HCl 0.3 M and buffered with 0.8 mL of alkaline borate buffer (30.9 g boric acid and 100 mL sodium hydroxide 1 M in 1 L of distilled water) and volume was adjusted to 10 ml with distilled water. 0.5 mL of a mix reagent (125 mL sulphuric acid 5 N, 37.5 mL ammonium molybdate saturated solution, 75 mL of ascorbic acid 0.1 M and 12.5 mL potassium antimonyl tartrate 1 mg/mL) were added to each tube. The absorbance of the solutions was measured at 882 nm.

The nitrogen and phosphorus requirements for growth (μmol N or P g^-1^ DW h^-1^) of the seedlings were calculated based on the growth rates (g DW g^-1^ DW day^-1^) measured *in situ* by Nielsen and Pedersen (2000) in *C. nodosa* seedlings from Alfacs Bay (NE Spain), multiplied by the total N and P content (%) of whole seedlings measured in the present study, assuming that N and P demand for leaf growth generally constitutes 95% of seagrass nutrient requirements (Erftemeijer et al., 1994; Stapel et al., 1996).

### Light and Fertilization Experiment

Seedlings were grown during 4 weeks in flasks with 0.2 L of autoclaved sea sand as physical substrate and 0.5 L of artificial seawater (salinity = 35‰, pH = 8.2) containing nitrogen and phosphorus at three different ratios of molar concentrations (40 μM N:10 μM P, 25 μM N:25 μM P and 10 μM N:40 μM P) at two light intensities (20 and 200 μmol quanta m^-2^ s^-1^), in a total of six treatments (*n* = 3 replicates per treatment with 2 seedlings per replicate). Nutrient ratios corresponded respectively to concentrations of 40 μM of NH_4_Cl:10 μM K_2_HPO_4_, 25 μM of NH_4_Cl:25 μM K_2_HPO_4_ and 10 μM of NH_4_Cl:40 μM K_2_HPO_4_. These concentrations were selected to be 4 to 10-fold higher than ambient concentrations (e.g., Pérez and Romero, 1994) to represent a fertilization scenario for restoration. Ammonium was chosen because it is the preferential inorganic nitrogen source for most seagrasses (Touchette and Burkholder, 2000; Alexandre et al., 2011, 2014). A light intensity of 200 μmol quanta m^-2^ s^-1^ was chosen because it is above the compensation and saturation irradiance for *C. nodosa* (Olesen et al., 2002; Olivé et al., 2013; Silva et al., 2013). A light intensity of 20 μmol quanta m^-2^ s^-1^ was chosen to simulate an accentuated light reduction. This value was determined based on the percentage of light attenuation (10% of the ambient surface irradiance) experienced by *C. nodosa* plants in a shallow (mean depth 3.2 m) and relatively turbid (light attenuation coefficient, *k* = 0.57 m^-1^) bay in Spain (Duarte, 1991). Seedlings were grown at 22°C under a photoperiod of 12:12 h. Light intensities were achieved using shade screens. The growth media were changed twice every week to keep the initial levels of nutrients and oxygen.

Leaf growth rates were determined weekly by measuring the total length (from the sheath to the leaf tip) of all leaves of each shoot. This method was preferred over the classical punching method used to determine seagrass growth (Zieman, 1974) to avoid damaging the tissues of the narrow and fragile leaves of the seedlings. The growth rate was calculated by subtracting the total leaf length (TLL) at the beginning to the TLL after 1 week and dividing by the number of days. The photosynthetic performance of the seedlings in each treatment was also measured weekly. The 2nd or 3rd youngest leaf of each seedling was irradiated with a series of eight increasing light intensities (1, 11, 22, 65, 88, 145, 217, and 294 μmol quanta m^-2^ s^-1^) using a pulse amplitude modulated (PAM) fluorometer (Diving PAM, Walz, Germany). The relative electron transport rate (rETR = Y × I μmol e^-^ m^-2^ s^-1^) was calculated for each irradiance step, where Y is the effective quantum yield and I is the irradiance (μmol quanta m^-2^ s^-1^). The effective quantum yield of photosystem II (Y) is determined as (F’_m_ - F_s_)/F’_m_, where F_s_ is the fluorescence in the light when only part of the reaction centers are closed and F_m_’ is the maximal fluorescence of a light adapted leaf immediately after closure of all reaction centers obtained through the application of a saturating light pulse (Genty et al., 1989). The rETR vs. irradiance curves were fitted to the model of Jassby and Platt (1976), rETR = rETR_m_ × tanh (α x I/rETR_m_), where I is irradiance, α is the ascending slope at limiting irradiances, and rETR_m_ is the relative maximum electron transport rate. Parameters were derived from curves with *R*^2^ > 0.82.

### Data Analysis

Differences in the nutrient content (C, N, and P) among non-germinated seeds and seedlings were detected with a *t*-test using SigmaPlot for windows Version 11.0 (Systat Software, Germany). The effects of light level, nutrient ratio and time of exposure on growth, rETR_m_ and alpha of the seedlings were tested with three-way analyses of variance with repeated measures (factors light and nutrient ratio between subjects; factor time within subject, repeated measure) using R programming language R 3.0.3 (R Core Team, 2014, R Foundation for Statistical Computing, Vienna, Austria). All tests were performed at a level of significance lower than 0.05.

## Results

### Germination of Seeds

**FIGURE 1:**
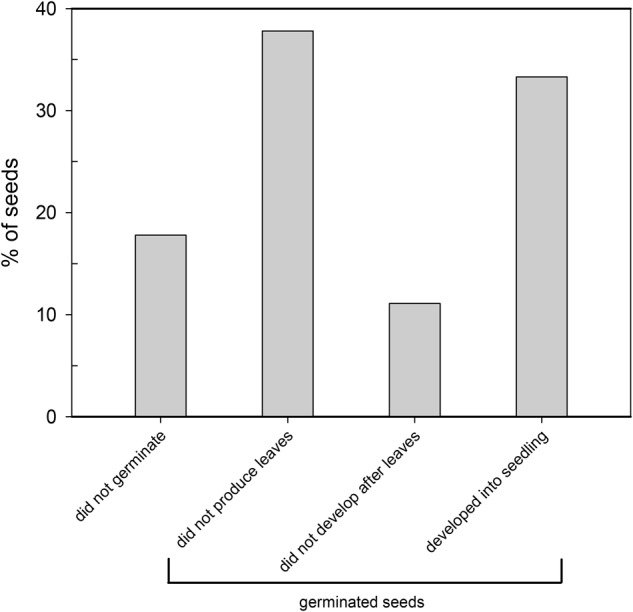
Percentage of *Cymodocea nodosa* seeds (*n* = 90) in each developmental stage, throughout a period of 48 days after seed germination was induced. Seeds were germinated in seawater (salinity of 18‰) at 22°C and light intensity of 150 μmol quanta m^-2^ s^-1^ under a 12 h:12 h light:dark cycle.

Eighty two percent of the seeds germinated after 48 days at a mean rate of 1.5 seeds per day. This indicates a high viability of the seeds used in our experiment. Germination was initially high on the second and fifth day (11 and 26%) and decreased afterward to values around 5% and lower. After 1 week, nearly 50% of the seeds had germinated. Of all seeds (*n* = 90), 18% did not germinate and only a small percentage (33%) reached the seedling stage. A similar percentage (38%) germinated but did not produce leaves and a small percentage of the seeds (11%) died after producing leaves (**Figure 1**).

### Uptake Experiment

**FIGURE 2:**
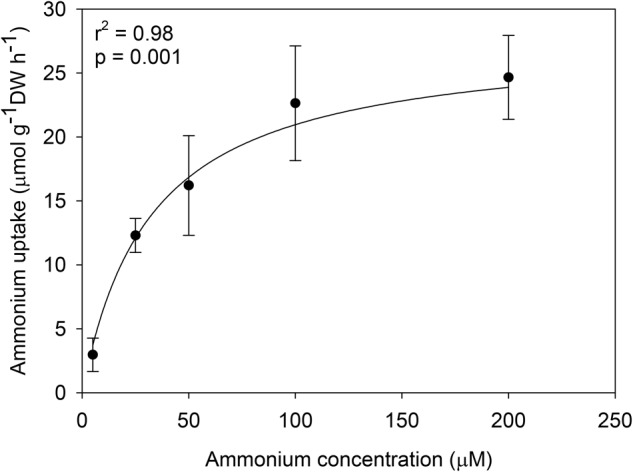
Ammonium uptake rates (μmol g^-1^ DW h^-1^) of *C. nodosa* seedlings as a function of nutrient concentration (μM). Data were fitted to the Michaelis–Menten model. The coefficient of determination (*r*^2^) and level of significance (*p*) of the fit are shown. Values are mean ± SD (*n* = 8).

**FIGURE 3:**
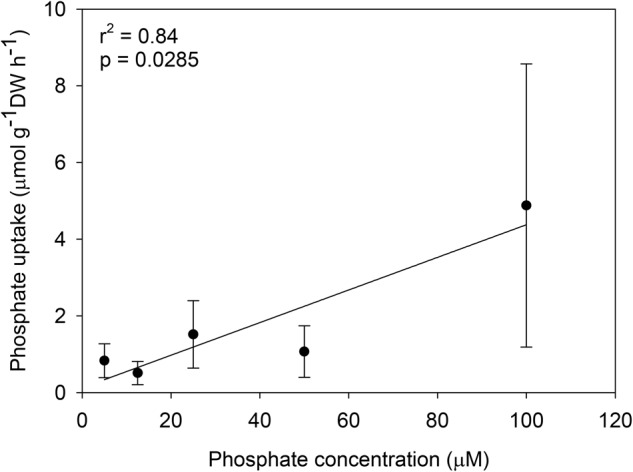
Phosphate uptake (μmol g^-1^ DW h^-1^) of *C. nodosa* seedlings as a function of nutrient concentration (μM). Data were fit to a linear model. The coefficient of determination (*r*^2^) and level of significance (*p*) of the fit are shown. Values are mean ± SD (*n* = 5).

The uptake of ammonium by *C. nodosa* seedlings followed a hyperbolic Michaelis–Menten kinetics (**Figure 2**). The maximum ammonium uptake rate (V_max_) of the seedlings was 27.74 μmol g^-1^ DW h^-1^, the half-saturation constant (K_m_) was 32.34 μM and the uptake affinity (α = V_max_/K_m_) was 0.86. In contrast, the uptake of phosphate by the seedlings showed no saturation kinetics and was best described by a linear regression model (V = 0.1223 + 0.0425 × S, *r*^2^ = 0.84) (**Figure 3**). Phosphate uptake was 0.83 μmol g^-1^ DW h^-1^ at 5 μM (3.5-fold lower than the ammonium uptake) and reached 4.88 μmol g^-1^ DW h^-1^ at 100 μM (5-fold lower than the ammonium uptake).

### Nutrient Content and Requirements for Growth

The carbon content was significantly different among non-germinated seeds and seedlings (*t* = 5.613, *p* = 0.001). Carbon content was higher in non-germinated seeds (42.06 ± 1.19%) and decreased significantly in the young seedlings (36.19 ± 1.72%). In contrast, the nitrogen content increased significantly from non-germinated seeds (1.40 ± 0.08%) to seedlings (2.23 ± 0.35%) (*t* = 4.675, *p* = 0.003). Consequently, the C:N ratio decreased significantly from non-germinated seeds (30.20 ± 1.03) to seedlings (16.41 ± 1.92). The phosphorus content was not significantly different between non-germinated seeds (0.41 ± 0.06%) and seedlings (0.47 ± 0.05%) (*t* = 1.797, *p* = 0.115). C:N:P ratio of non-germinated seeds was 105:4:1, whereas the ratio of the seedlings was 77:5:1. The nitrogen and phosphorus requirements for growth of *C. nodosa* seedlings were 3.12 μmol N g^-1^ DW h^-1^ (74.9 μmol N g^-1^ DW day^-1^) and 0.29 μmol P g^-1^ DW h^-1^ (6.9 μmol P g^-1^ DW day^-1^), respectively. These values were calculated based on a mean growth rate of 0.047 g DW g^-1^ DW day^-1^ measured by Nielsen and Pedersen (2000) in *C. nodosa* seedlings and considering the total content of nitrogen (2.23%) and phosphorus (0.47%) in the seedlings determined in the present study.

### Light and Fertilization Experiment

**FIGURE 4:**
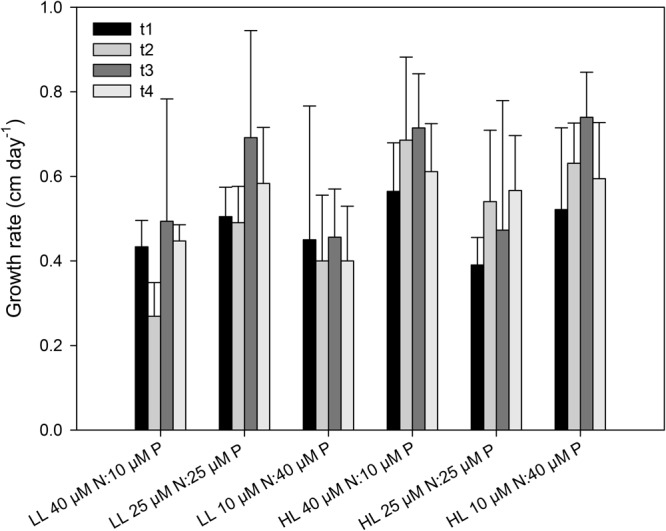
Growth rate (cm day^-1^) of *C. nodosa* seedlings incubated at different nitrogen to phosphate ratios (40 μM N:10 μM P, 25 μM N:25 μM P and 10 μM N:40 μM P) under low light (LL = 20 μmol quanta m^-2^ s^-1^) and high light (HL = 200 μmol quanta m^-2^ s^-1^) along the experiment (*t*_1_ = after 1 week; *t*_2_ = after 2 weeks; *t*_3_ = after 3 weeks; *t*_4_ = after 4 weeks). Values are mean ± SD (*n* = 6).

**FIGURE 5:**
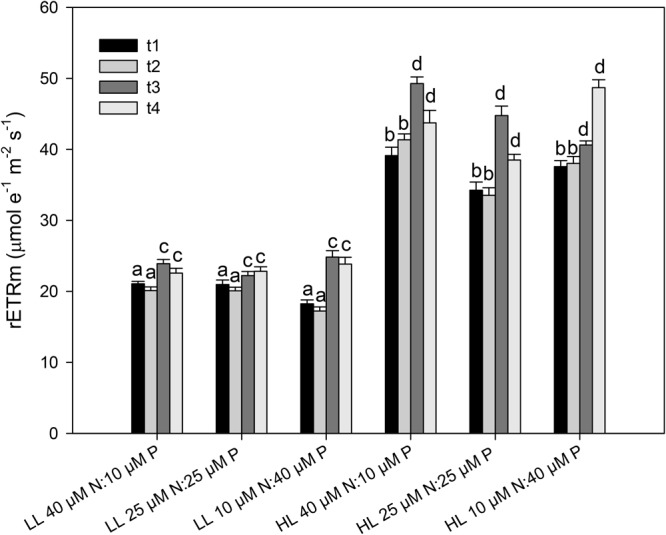
Maximum relative electron transport rate (rETR_m_) of *C. nodosa* seedlings incubated at different nitrogen to phosphate ratios (40 μM N:10 μM P, 25 μM N:25 μM P and 10 μM N:40 μM P) under low light (LL = 20 μmol quanta m^-2^ s^-1^) and high light (HL = 200 μmol quanta m^-2^ s^-1^) intensity along the experiment (*t*_1_ = after 1 week; *t*_2_ = after 2 weeks; *t*_3_ = after 3 weeks; *t*_4_ = after 4 weeks). Values are mean ± SD (*n* = 6).

**FIGURE 6:**
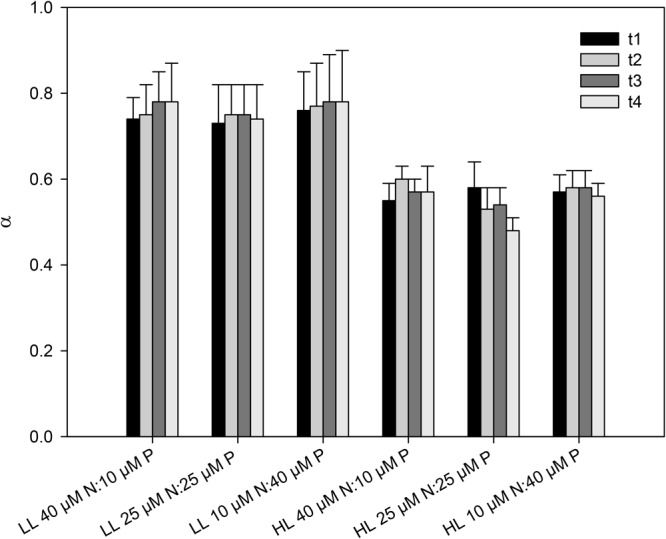
Initial slopes (α = μmol e^-^ m^-2^ s^-1^/μmol quanta m^-2^ s^-1^) of *C. nodosa* seedlings incubated at different nitrogen to phosphate ratios (40 μM N:10 μM P, 25 μM N:25 μM P and 10 μM N:40 μM P) under low light (LL = 20 μmol quanta m^-2^ s^-1^) and high light (HL = 200 μmol quanta m^-2^ s^-1^) intensity along the experiment (*t*_1_ = after 1 week; *t*_2_ = after 2 weeks; *t*_3_ = after 3 weeks; *t*_4_ = after 4 weeks). Values are mean ± SD (*n* = 6).

The growth rate of the seedlings increased significantly with light (*p* = 0.04) (**Figure 4** and **Table 1**). On average, seedlings exposed to high light (200 μmol quanta m^-2^ s^-1^) grew 20% more than seedlings exposed to a low light (20 μmol quanta m^-2^ s^-1^). In contrast, growth rates were not significantly affected by the nutrient ratio (*p* = 0.996) or time of exposure (*p* = 0.051), and no interactions among factors were detected (**Table 1**). None of the seedlings died during the 4 weeks of the experiment. rETR_m_ and α were significantly affected by the light level (*p* < 0.001) but not by nutrient ratios (**Figures 5, 6** and **Table 1**). On average, rETR_m_ values of seedlings exposed to high light were twice as high as those of seedlings exposed to low light, while alpha values were about 25% lower. The time of exposure had a significant effect on the rETR_m_ of the seedlings (*p* < 0.001), but not on α (*p* = 0.355). rETR_m_ increased significantly from the 2nd to the 3rd week of experiment, particularly in the high light treatment but this difference disappeared after the 3rd week.

**Table 1:** Combined effects of light level, nutrient ratio and time of exposure on growth, maximum electron transport rates (rETR_m_) and α of *Cymodocea nodosa* seedlings, as determined by three-way analysis of variance with repeated measures with a level of significance < 0.05 (values in bolt denote significant effects).

	df	*F*	*p*
***Growth***			
Light	1	4.788	**0.037**
Nutrient ratio	2	0.004	0.996
Time	3	2.697	0.051
Light × Nutrient ratio	2	3.252	0.053
Light × Time	3	1.827	0.148
Nutrient ratio × Time	6	0.319	0.925
Light × Nutrient ratio x Time	6	0.702	0.649
***rETR_m_***			
Light	1	323.282	**<0.001**
Nutrient ratio	2	3.344	0.051
Time	3	11.605	**<0.001**
Light × Nutrient ratio	2	2.266	0.123
Light × Time	3	1.297	0.281
Nutrient ratio × Time	6	0.919	0.485
Light × Nutrient ratio × Time	6	1.312	0.260
***α***			
Light	1	166.639	**<0.001**
Nutrient ratio	2	1.682	0.205
Time	3	1.097	0.355
Light × Nutrient ratio	2	0.230	0.796
Light × Time	3	1.735	0.166
Nutrient ratio × Time	6	1.173	0.329
Light × Nutrient ratio × Time	6	0.838	0.544

## Discussion

Following the protocol developed by Zarranz et al. (2010) we were able to obtain germination rates of *Cymodocea nodosa* seeds similar to those they reported (82% vs. 50 – 90%), but a smaller percentage of seeds reaching the seedling stage (33% compared to 42 – 85%). In both studies, the seeds were collected from the sediment rather than directly from reproductive shoots, and therefore it is not possible to know how putative different residence times within the sediment may affect seed reserves and germination rates. The collection of seeds directly from reproductive shoots rather than from the sediment may be an alternative to improve seed germination and seedling development rates. The seed germination rates observed for *C. nodosa* are much higher than those reported for the seagrass *Z. marina* (generally 10% or less, Orth et al., 2003).

*Cymodocea nodosa* seedlings acquired nutrients from the water while still attached to the seed and exhibited distinct uptake kinetics for ammonium and phosphate. The uptake of ammonium followed saturation kinetics whereas the uptake of phosphate increased linearly with nutrient concentration within the range tested (5 – 100 μM). The uptake rate of ammonium by *C. nodosa* seedlings were about twice as the uptake rate of adult plants of *C. nodosa* (4.10 ± 1.99 μmol g^-1^ h^-1^) measured by Morris et al. (2013) using a similar incubation time (0.67 h) and at *in situ* ammonium concentration of 11 μM at a 3-m depth meadow in Cadiz Bay. The affinity constant (α = 0.86) for the ammonium uptake is among the highest reported for seagrass species (cf. Alexandre et al., 2011 and references therein) and indicates that *C. nodosa* seedlings can effectively acquire this nutrient when present at low concentrations in the environment. The difference in the slopes of the uptake kinetics of nitrogen (α = 0.86) and phosphorus (α = 0.04) suggests that the seedlings acquire nitrogen from the external medium more efficiently than phosphorus. Phosphorus uptake rates fell within the range of reported values (0.5 – 3 μmol g^-1^ h^-1^ for phosphate concentrations between 5 and 25 μM) for leaves of adult plants of other seagrass species (Pérez-Lloréns and Niell (1995) for *Zostera noltii*; Gras et al. (2003) for *Thalassia testudinum*; Nayar (2015) for *Amphibolis antarctica* and *Posidonia angustifolia*) but were slightly lower when compared to seedlings of *A. antarctica* from Western Australia (0.8 μmol g^-1^ DW h^-1^ vs. 2 μmol g^-1^ DW h^-1^ at 5 μM) (Paling and McComb, 1994).

The ability of *C. nodosa* young seedlings to take up nutrients from the surrounding environment is a feature that may contribute to successful seedling-based restoration of this species if compared, for example, to *P. oceanica*, where inadequate nutrient uptake of the seedlings due to small root systems has been pointed out as a major cause of transplant failure (Balestri and Bertini, 2003; Lepoint et al., 2004). On the other hand, additions of N and P to seagrass sediments constrained root development (root mass, length, and density of lateral root branches) in the early stages of seedling growth in *P. australis*, hindering the capacity for successful anchorage of the seedlings (Statton et al., 2014). In this species, the seedlings’ growth and root development were also tightly coupled with the type of the sediment and the addition of organic matter. The effect of these and other important environmental stressors such as temperature and sediment burial, and their interactions, must be assessed in future studies of *C. nodosa* seedlings in order to improve the selection of suitable sites for seedling-based restoration of this species.

The leaf and root tissues of the seedlings were richer in nitrogen when compared to the endosperm of non-germinated seeds. This indicates that young seedlings (∼1 month old) of *C. nodosa* are able to use nitrogen from the external environment in the early stage of their development. A similar pattern of increasing nutrient content from seed (0.02 mg P seed^-1^ and 0.32 mg N seed^-1^, Duarte and Sand-Jensen, 1996) to seedling [38.7 mg P seedling^-1^ and 542 mg N seedling^-1^; Nielsen and Pedersen (2000) has been previously reported for *C. nodosa*]. This nutrient acquisition strategy differs from that of *P. australis* and other aquatic plants (e.g., maize or rice), whose seedlings were found to be dependent, on seed reserves rather than external nutrient sources until these are exhausted, and only after that, roots develop their uptake capacity (Statton et al., 2014; Cheng et al., 2015; Sabermanesh et al., 2017).

In our experiment, the seawater used in the aquaria with the seeds contained nitrogen and phosphorus at concentrations that varied between 1 and 5 μM. Based on these concentrations and the uptake results, we estimate that the uptake of nitrogen and phosphorus by the seedlings was in the range 14 – 71 μmol N g^-1^ DW day^-1^ and 4 – 20 μmol P g^-1^ DW day^-1^, respectively. This nutrient acquisition would meet the requirements for growth of the seedlings in terms of phosphorus (7 μmol P g^-1^ DW day^-1^) but not nitrogen (75 μmol N g^-1^ DW day^-1^), but these estimations account for inorganic nitrogen only (as ammonium). In addition to ammonium, seagrasses are able to take up nitrate and organic nitrogen (Alexandre et al., 2011, 2015), and therefore the nitrogen supply from these sources must be also added to the nitrogen budget. The above calculations further support our findings that young seedlings of *C. nodosa* use external nutrient sources when available to meet their nutrient requirements for growth, probably as a resource management strategy to save internal seed reserves to maintain growth and increase the resilience in environments where nitrogen and phosphorus concentrations are limiting, such as in most seagrass meadows (Duarte and Sand-Jensen, 1996; Nielsen and Pedersen, 2000). Seed reserves may be useful in a later stage of development (Kennedy et al., 2004; Thangapandian et al., 2006).

Light availability in shallow coastal environments can be highly variable, mostly due to dissolved and particulate substances in the water column. Consequently, the amount of light reaching seagrasses at the bottom can be markedly attenuated (10% of the ambient surface irradiance, Duarte, 1991). Our results showed that light was more important for seedlings’ growth than the nutrient ratio. Increasing the light available to the seedlings resulted in an increase of growth (as leaf elongation rate) of 20%, whereas different nitrogen to phosphorus molar ratios did not affect growth significantly within the 4 weeks of the experiment. The availability of light also affected the photosynthetic activity of the young seedlings. The maximum relative photosynthetic capacity (rETR_m_) under high light was twice of that measured under the low light treatment whereas the photosynthetic efficiency (α) decreased by 25%, a pattern that persisted until the end of the experiment. Based on these results, it is recommended that seedlings of *C. nodosa* be planted in shallow depth areas where light levels are expected to be higher. Shallow areas should be, however, sheltered from wave dynamics as strong wave exposure and sediment mobility reduce transplant survival (de Jonge et al., 2000; Bos and van Katwijk, 2007).

Our results showed that seedlings of *C. nodosa* have the ability to rapidly acclimate to the surrounding light environment. Natural stands of adult *C. nodosa* plants showed a similar photoacclimatory response of reduced maximum photosynthetic rates and improved photosynthetic efficiency when exposed to different levels of light attenuation (24, 40, and 75% of naturally available photosynthetic active radiation = 228, 180, and 75 μmol quanta m^-2^ s^-1^, respectively) in a 3-week shading experiment (Silva et al., 2013). Under light deprivation, adult plants rapidly re-arranged their photosynthetic pigment pool, adjusting to a pattern typical of shade acclimated leaves, a response which is probably related to the photoacclimatory and pigmentary plasticity of the species (Olivé et al., 2013), and used both soluble sugars and starch stored in the leaves to maintain metabolic processes. Severe light limitation was also shown to reduce survival, growth, size and biomass of *Zostera marina* seedlings. Under a 90% reduction of incident light, growth and belowground biomass decreased by 80% and the production of lateral shoots ceased, compromising the long-term survival of the seedlings and the development of new meadows (Bintz and Nixon, 2001).

Experimental nutrient additions resulted in ammonium and phosphate levels slightly above those typically found in seagrass habitats (Touchette and Burkholder, 2000), which are often limiting for seedling growth. The experimental concentration of ammonium (40 μM) is close to that used previously in other ammonium enrichment experiments (∼30 μM) (e.g., van Katwijk et al., 1997; Brun et al., 2002; Villazán et al., 2016). In the field, nutrient additions to seagrass meadows are usually made through the application of fertilizers with either balanced or unbalanced nutrient formulations. Therefore, the nutrient ratios used in our study were chosen to mimic those of common commercial fertilizers. These are slow-release fertilizers, which means that nutrients are released to the surrounding area at slow rates, in contrast to eutrophication areas where nutrients enter the systems at very high rates and concentrations (Cloern, 2001). The application of different nutrient formulations (balanced and unbalanced N and P ratios) did not affect significantly the growth of the seedlings. Therefore, our initial hypothesis that seedling growth would be higher under high light with a balanced formulation was not verified. The lack of effect may be related to the possible use of nutrient reserves by the seedlings still attached to the seed while using also nutrients from the external environment. However, seedlings grown under the low light level consistently showed slightly higher growth rates when nourished with a balanced nutrient ratio (25 μM N:25 μM P) compared with those receiving unbalanced formulations, as was also reported by Roberts et al. (1984) and Tanner and Parham (2010) for seedlings of *Z. marina*. The rapid entry of ammonium into the cells causes a series of complex physiological alterations such as the uncoupling of ATP production and photosynthetic electron transport, enhanced respiratory demands and depletion of essential cations (potassium, magnesium, calcium) (cf. Villazán et al., 2013 and references therein). Seagrasses minimize these effects through rapid assimilation of ammonium and conversion into amino acids, which generates a strong depletion of carbon skeletons (Brun et al., 2002, 2008). The decrease in C content observed in the seedlings relative to the seeds may be explained by this carbon use. The assimilation of ammonium under light deprivation requires an even greater energy consumption because carbon reserves are mostly restored through photosynthesis, and often required mobilization of carbohydrates. However, the side effects of high-rate ammonium assimilation may be ameliorated in the presence of phosphate (Brun et al., 2008), whereas the presence of ammonium affects negatively the uptake rate of phosphate (Villazán et al., 2013). These observations suggest that in the unbalanced 40 μM N:10 μM P formulation, the growth of *C. nodosa* seedlings under low light was hampered by the combination of insufficient carbon reserves and lower phosphate uptake. Under low light, the balanced formulation resulted in a faster growth of the seedlings, probably because of the higher ammonium availability (25 μM) relatively to the 10 μM N:40 μM P treatment (10 μM). Under high light, the seedlings generally displayed slightly lower growth rates when supplied with the 25 μM N:25 μM P nutrient treatment, suggesting that the decrease in the growth rate is related to the generally lower photosynthetic activity (rETR) of the seedlings in this treatment (HL 25 μM N:25 μM P).

## Conclusion

Our study showed that light was more important than water column nutrients for the successful development of young seedlings of *C. nodosa*. From a restoration perspective, the use of seedlings to enhance the recovery or genetic diversity of *C. nodosa* meadows will thus be more efficient in shallow areas with high light levels. Seedlings still attached to seeds obtain nutrients from the water at higher rates than adult plants. The relevance of external nutrient uptake was further highlighted by the observation that seedlings’ tissues were richer in nitrogen and phosphorus than non-germinated seeds. This shows that seedlings of *C. nodosa* have the ability to rapidly acclimate to the surrounding environment while still attached to the seeds. The use of balanced vs. unbalanced nutrient fertilization to obtain higher seedling growth depends on the light level to which seedlings are exposed. Seedlings experiencing low light levels showed slightly enhanced growth if nourished with a balanced formulation, whereas a slight increase in growth was observed with an unbalanced formulation under a higher light level.

## Author Contributions

AA and RS conceived and designed the study. AA and JS carried out the experimental work. AA analyzed the data. AA, JS, and RS wrote the manuscript and gave final approval.

## Conflict of Interest Statement

The authors declare that the research was conducted in the absence of any commercial or financial relationships that could be construed as a potential conflict of interest.
